# A Refined Study of *FCRL* Genes from a Genome-Wide Association Study for Graves’ Disease

**DOI:** 10.1371/journal.pone.0057758

**Published:** 2013-03-07

**Authors:** Shuang-Xia Zhao, Wei Liu, Ming Zhan, Zhi-Yi Song, Shao-Ying Yang, Li-Qiong Xue, Chun-Ming Pan, Zhao-Hui Gu, Bing-Li Liu, Hai-Ning Wang, Liming Liang, Jun Liang, Xiao-Mei Zhang, Guo-Yue Yuan, Chang-Gui Li, Ming-Dao Chen, Jia-Lun Chen, Guan-Qi Gao, Huai-Dong Song, The China Consortium for the Genetics of Autoimmune Thyroid Disease

**Affiliations:** 1 State Key Laboratory of Medical Genomics, Molecular Medicine Center, Ruijin Hospital Affiliated to Shanghai Jiaotong University (SJTU) School of Medicine, Shanghai, China; 2 Department of Endocrinology, Shanghai Institute of Endocrinology and Metabolism, Ruijin Hospital Affiliated to SJTU School of Medicine, Shanghai, China; 3 Shanghai Center for Systems Biomedicine, SJTU, Shanghai, China; 4 Department of Epidemiology and Biostatistics, Harvard School of Public Health, Boston, Massachusetts, United States of America; 5 Department of Endocrinology, The Central Hospital of Xuzhou Affiliated to Xuzhou Medical College, Xuzhou, Jiangsu Province, China; 6 Department of Endocrinology, The First Hospital Affiliated to Bengbu Medical College, Bengbu, Anhui Province, China; 7 Department of Endocrinology, The Hospital Affiliated to Jiangsu University, Zhenjiang, Jiangsu Province, China; 8 Department of Endocrinology, Gout Laboratory, Medical School Hospital of Qingdao University, Qingdao, Shandong Province, China; 9 Department of Endocrinology, Linyi People’s Hospital, Linyi, Shandong Province, China; University of Michigan, United States of America

## Abstract

To pinpoint the exact location of the etiological variant/s present at 1q21.1 harboring *FCRL1-5* and *CD5L* genes, we carried out a refined association study in the entire *FCRL* region in 1,536 patients with Graves’ disease (GD) and 1,516 sex-matched controls by imputation analysis, logistic regression, and cis-eQTL analysis. Among 516 SNPs with *P*<0.05 in the initial GWAS scan, the strongest signals associated with GD and correlated to *FCRL3* expression were located at a cluster of SNPs including rs7528684 and rs3761959. And the allele-specific effects for rs3761959 and rs7528684 on *FCRL3* expression level revealed that the risk alleles A of rs3761959 and C of rs7528684 were correlated with the elevated expression level of *FCRL3* whether in PBMCs or its subsets, especially in CD19^+^ B cells and CD8^+^ T subsets. Next, the combined analysis with 5,300 GD cases and 4,916 control individuals confirmed *FCRL3* was a susceptibility gene of GD in Chinese Han populations, and rs3761959 and rs7528684 met the genome-wide association significance level (*P_combined_* = 2.27×10^−12^ and 7.11×10^−13^, respectively). Moreover, the haplotypes with the risk allele A of rs3761959 and risk allele C of rs7528684 were associated with GD risk. Finally, our epigenetic analysis suggested the disease-associated C allele of rs7528684 increased affinity for NF-KB transcription factor. Above data indicated that *FCRL3* gene and its proxy SNP rs7528684 may be involved in the pathogenesis of GD by excessive inhibiting B cell receptor signaling and the impairment of suppressing function of Tregs.

## Introduction

Graves’ disease (GD), the most frequent form of autoimmune thyroid disease (AITD), is triggered by the combination of genetic susceptibility and environmental encounters. Using the candidate gene strategy, several susceptibility genes for GD have been validated in different ethnic populations and have been divided into two classes: one class is immune-related genes, such as *HLA* on 6p21 [1], [2], *CTLA-4* on 2q33 [3], [4], *CD40* on 20q12 [5], [6], *PTPN22* on 1p13 [7]–[9], as well as *SCGB3A2* on 5q31 [10]–[12]; and the other class is thyroid-specific gene, such as *TSHR* on 14q31 [13], [14].

*FC receptor-like-3* (*FCRL3,* also known as CD307c) on 1q21.1 encodes a member of the immunoglobulin receptor superfamily and is one of several Fc receptor-like glycoproteins. The encoded protein of *FCRL3* contains immunoreceptor-tyrosine activation motifs and immunoreceptor-tyrosine inhibitory motifs in its cytoplasmic domain and may play a role in regulation of the immune system. The 1p21–23 region, in which the *FCRL* family resides, has been identified as a candidate locus for multiple autoimmune disorders in both human and murine models [15]. Mutations in *FCRL3* have been reported to be associated with a plethora of autoimmune diseases, such as rheumatoid arthritis, systemic lupus erythematosus, and AITD [16]–[18]. Recently, two genome-wide association studies (GWAS) from Wellcome Trust Case Control Consortium (WTCCC) and our group, both identified *FCRL3* as a susceptibility gene of GD in individuals of European ancestry and Chinese Han populations, respectively [19], [20]. After the WTCCC GWAS, a case–control association study investigating twelve tag SNPs within *FCRL5* was performed in 2,504 UK Caucasian patients with GD and 2,688 geographically matched controls and the results suggested that the association of *FCRL5* with GD is secondary to the effect of *FCRL*3 [21]. Nevertheless, a refined association study in the entire *FCRL* region is required to determine the exact location of the etiological variant/s present.

In this study, we refined the association in the 1q21.1 region harborbing *FCRL1-5* and *CD5L*, and confirmed *FCRL3* was a susceptibility gene of GD in Chinese Han populations and the most significant signals associated with GD and correlated to *FCRL3* expression were located at a cluster of SNPs including rs3761959 and rs7528684. Moreover, the haplotypes with the risk allele A of rs3761959 and the risk allele C of rs7528684 were associated with the predisposition of GD and can up-regulate the mRNA expression level of *FCRL3*, whether in peripheral blood mononuclear cells (PBMCs) or the subsets of PBMCs, especially in CD19^+^ B cells and CD8^+^ T subsets. Finally, the risk allele C of rs7528684 can increase the binding with NF-KB transcription factor, resulting in the pathogenesis of GD.

## Materials and Methods

### Subjects

All samples were recruited from Chinese Han population through collaboration with multiple hospitals in China. This study was approved by the local ethics committee from Ruijin Hospital, the Central Hospital of Xuzhou, the first affiliated hospital of Bengbu Medical College, Medical School Hospital of Qingdao University, and Linyi People’s Hospital, respectively. And all subjects in this study provided written informed consent using protocols approved by local ethics committee. As mentioned in our previous GWAS paper, 1,536 patients with GD and 1,516 sex-matched controls were recruited for the initial GWAS stage, and additional 3,994 patients with GD and 3,510 sex-matched controls were recruited for the replication study [4], [10], [20]. Diagnosis of GD was based on documented clinical and biochemical evidence of hyperthyroidism, diffuse goiter, and the presence of at least one of the following: positive TRAb tests, diffusely increased ^131^I uptake in thyroid gland, or exophthalmos [4], [10], [20]. All individuals classified as GD were interviewed and examined by experienced clinicians.

All the 1,516 controls in the GWAS stage were individuals with neither GD nor family history of GD, and without any other autoimmune disorders. Control subjects were matched for sex with cases and were over 35 years. Since GD or other AITD has a preponderance in the young female population, this age criteria could reduce the number of controls who might develop GD later on. To exclude clinical or sub-clinical AITD, the levels of sensitive TSH (sTSH) and TPOAb in control subjects were measured using chemiluminescence immunoassay (CLIA) in our laboratory. Of the 1,832 healthy controls whose levels of sensitive TSH and TPOAb were measured, 257 individuals with the levels of TPOAb ≥5.61 U/ml, and 94 subjects with the levels of sensitive TSH ≥4.94 µU/ml or ≤0.35 µU/ml were excluded, the remaining 1,516 served as the control cohort in the GWAS stage [20].

### Genotyping and Quality Control

GWAS was performed by Illumina Human660-Quad BeadChips [20]. Genotype clustering was conducted using Illumina GenomeStudio V2011.1 software based on the 660W-Quad_v1_H manifest files. This software, which is used to convert the fluorescence intensities into SNP genotypes, was different from the software used in the previously published GWAS paper [20]. The mean call rate across all samples was 99.8%. Quality filtering was performed on SNPs and samples before analysis to ensure robust association tests. Cryptic relationships between genotyped individuals were examined using pairwise identity-by-descent (IBD) estimation by PLINK software [20]. To maintain the maximum number of available samples, all the pairwise relationships were evaluated and the person who formed the node that related to the most other nodes in the family trees was first excluded. This process was iterated several times until the remaining samples were not related to one another.

Of the 655,214 markers assayed, 3,185 that were from the Y or mitochondrial chromosomes or were CNV-related were excluded. Next, 168,082 markers with Hardy-Weinberg equilibrium *P*≤10^−6^, with genotype call rates below 98%, or with a minor allele frequency (MAF) <0.01 were discarded, leaving 483,947 SNPs for subsequent analysis. After removing samples with low call rates (<98%, n = 23), gender inconsistencies (n = 6), and cryptic relatedness (n = 113), 2,910 samples were available for further association analysis.

In the replication cohort, six SNPs on 1q21.1 were genotyped using TaqMan SNP Genotyping Assays in Fludigm EP1 platform [20], and one SNP (rs7528684) was genotyped using ABI 7900HT platform. Of the seven SNPs genotyped, none of SNPs was removed for further association analysis. Ultimately, 3,655 GD patients and 3,385 controls with a 100% call rate were analyzed in the replication cohorts.

### Statistical Analysis

After quality control [20], we used the genotypes of 67 SNPs on 1q21.1 in 1,442 patients with GD and 1,468 controls for association analysis using the Cochran-Armitage trend test by PLINK [22]. The forward and two locus logistic regression analysis were performed using R statistics packages. The linkage disequilibrium (LD) block was analyzed by Haploview software version 4.2.

The genotype imputation was performed using IMPUTE2 software [23] and the updated 1000G phase 1 integrated variant set (Mar 2012) were used as a reference. Of the imputed SNPs, we analyzed only those that could be imputed with a relatively high confidence (estimated probability >0.9), had a MAF >1%, a genotype call rate >98%, and a Hardy-Weinberg equilibrium *P*-value >10^−6^. To take into account the uncertainty of imputed SNPs, the association analysis of the imputed SNPs was carried out utilizing the SNPTEST v2 software [24].

We inspected three eQTL databases from European Caucasian population. One was developed by Dixon et al. and contained 405 children of British descent organized into 206 sibships including 297 sib pairs and 11 half-sib pairs [25]. Another database assessed the transcriptome of circulating monocytes from 1,490 German individuals [26]. The third database was the cell type–specific eQTLs relevant to immunity and inflammation in paired samples of primary monocytes and B cells, purified by positive selection directly from 283 healthy British individuals [27].

For the replication stage, the Cochran-Armitage test for trend was used to examine the associations. Association analysis in the combined samples was performed by Cochran-Mantel-Hanezel stratification analysis [22]. We examined heterogeneity among studies using the Breslow-Day test [22], [28]. The genome-wide significance level was set at 5×10^−8^, in keeping with the current consensus of the field.

### Real-time RT-PCR

Blood samples (10 ml) were collected from 95 unrelated healthy Chinese Han volunteers for gene expression analysis in PBMCs. Samples with more blood volume (100 ml) were donated by these 95 individuals for gene expression assay in distinct subpopulations of PBMCs. The genotypes of rs761959 and rs7528684 among these 95 individuals were determined using the ABI 7900HT System (AA and TT, n = 29; AG and TC, n = 47; and GG and CC, n = 19). The CD4^+^, CD8^+^, CD14^+^ and CD19^+^ subsets of PBMCs were isolated using MACS Column kits (Miltenyi Biotec) according to the manufacturer’s instructions. The purity of each cell subpopulation was determined by an LSR II Flow Cytometer (BD Biosciences), and the cell subpopulations with the purity of over 90% were used for real time RT-PCR [20]. The mean purities of the CD4^+^, CD8^+^, CD14^+^ and CD19^+^ subsets were 98.3±1.7%, 98.3±1.7%, 96.9±1.9% and 96.2±2.5%, respectively [20]. cDNAs were made from 1 µg RNA templates using reverse transcriptase and oligo(dT) primer (Promega). Quantitative RT-PCRs for *FCRL3* at 1q21.1 were performed in duplicate using the SYBR Green and an ABI 7900HT Fast Real-Time PCR System. Expression of all samples was normalized to the relative expression level of *GAPDH*. Primer sequences for real-time PCR were as follows: human *FCRL3* primers (forward, 5′ TGGAGATAGCAACCCGATTTATTC 3′; reverse, 5′ CTGTAAGTTCCTCATGCTCTTGATG 3′) and *GAPDH* (forward, 5′ GAAGGTGAAGGTCGGAGTC 3′; reverse, 5′ GAAGATGGTGATGGGATTTC 3′). We performed statistical analysis for expression data using ANOVA and an unpaired Student’s *t*-test (the two tail *P* value is indicated on the figures).

## Results

The association and cis-gene expression (cis-eQTL) analysis in the initial GWAS scan cohort.

**Figure 1:**
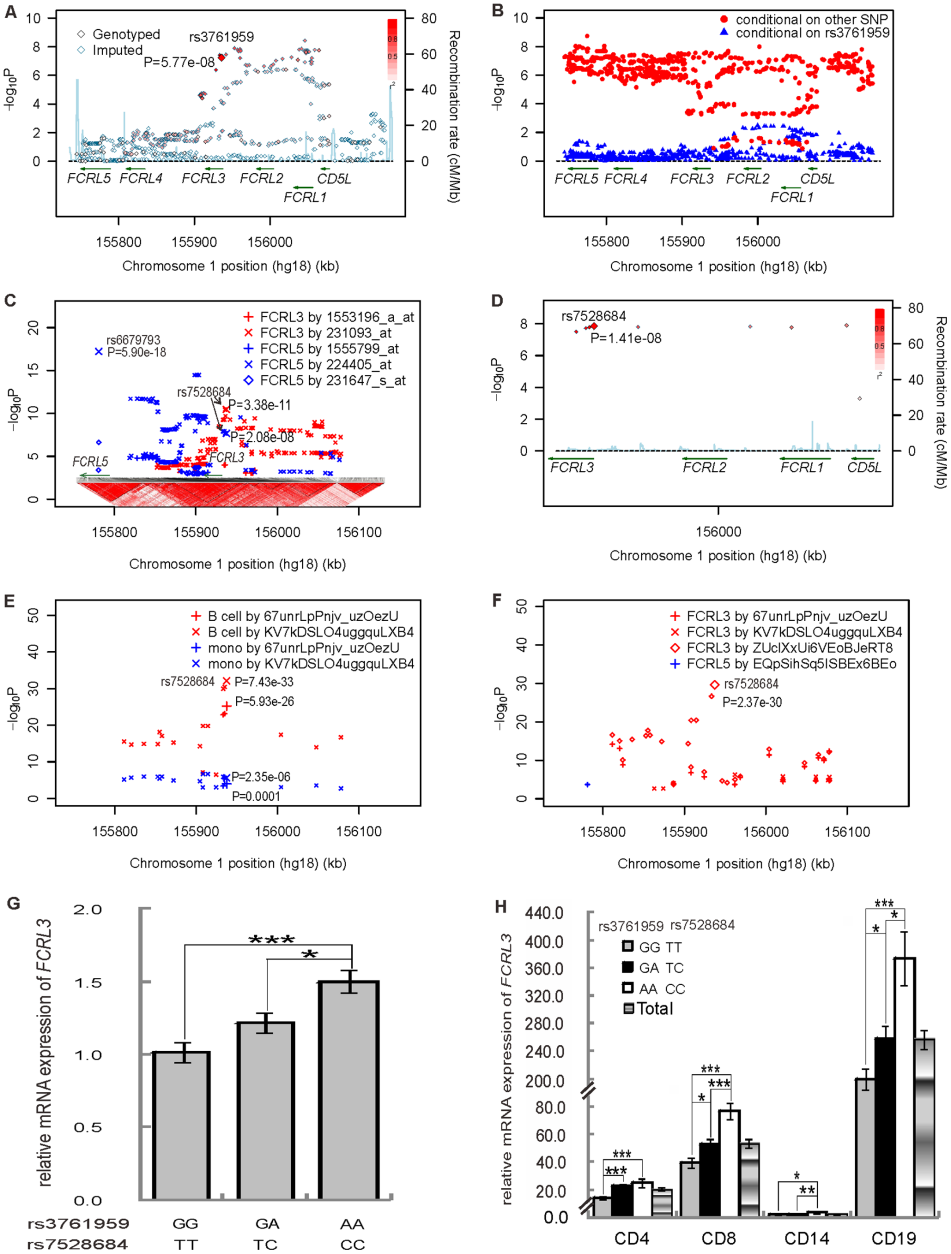
Regional plots of association results, logistic regression analysis, and cis-eQTL analysis at 1q21.1 and expression analysis of *FCRL3*. Panel A shows the GD association of 67 genotyped and 972 imputed SNPs in the GWAS samples. The color of each genotyped SNP spot reflects its r^2^ with the top SNP within each association locus shown as a large red diamond, and smaller values changing from red to white. Genetic recombination rates are shown in cyan. Genetic recombination rates, estimated using the 1000 Genomes pilot 1 CHB and JPT samples, are showing cyan. Physical positions are based on NCBI build 36. Panel B shows the two locus logistic regression analysis results for 1,039 SNPs at 1q21.1 in the GWAS samples. The SNPs were improved by adding rs3761959 were shown in red points; whileas, the SNPs improved the model with rs3761959 were showed in blue triangles. Panels C,D,E, and F show the cis-eQTL analysis of 516 SNPs with *P*<0.05 in the initial GWAS scan from three different cis-eQTL databases. Panel C shows plot of linkage disequilibrium (LD) structures at 1q21.1 and correlation of SNPs to transcript abundances of *FCRL3* and *FCRL5* genes at 1q21.1 in transcriptome data from about 400 lymphoblastoid cell lines. The LD structures of 1,039 SNPs at 1q21.1 were analyzed by Haploview software version 4.2 based on our imputed data. The LD color scheme is stratified according to the logarithm of the odds (LOD) score and D′: LOD <2 (white for D′ <1 and blue for D′ = 1) or LOD >2 (shades of pink/red for D′ <1 and bright red for D′ = 1). Two different red crosses indicate the association results of SNPs to the expression level of *FCRL3*. Three different blue signs indicate the correlation results of SNPs to the expression level of *FCRL5*. Panel D shows the correlation of 10 SNPs to transcript abundances of *FCRL3* in the transcriptome of circulating monocytes from 1,490 individuals. Panel E shows the correlation of SNPs to the expression of *FCRL3* in both-cis dataset of the cell type–specific cis-eQTL database. The expression levels of *FCRL3* in B cell and monocyte were shown in red and blue color, respectively. *FCRL3* expression detected by different probe was shown in two different crosses. Panel F shows the correlation of SNPs to the expression of *FCRL3* and *FCRL5* in B-cis dataset of the cell type–specific cis-eQTL database. The expression of *FCRL3* was shown in different red signs and that of *FCRL5* was shown in blue. Panel G shows the relative mRNA expression levels of *FCRL3* for different genotypes of rs3761959 and rs7528684 in PBMCs from 95 individuals (GG and TT, n = 29; GA and TC, n = 47; and AA and CC, n = 19). Panel H shows the relative mRNA expression levels of *FCRL3* for different genotypes of rs3761959 and rs7528684 in the subset of PBMCs from above 95 individuals. *, *P*<0.05; **, *P*<0.01; ***, *P*<0.001.

Our previous two-staged GWAS illustrated that *FCRL3* was a predisposing gene of GD on 1q21.1 harboring *FCRL1-5* and *CD5L* genes [20]. To pinpoint the exact location of the etiological variant/s present, we carried out the imputation analysis in the initial scan cohorts with 1,442 affected individuals and 1,468 controls. Within the ∼414-kb region of high LD at 1q21.1, there were 67 genotyped SNPs and 972 imputed SNPs (Figure 1A, Table S1). The mean variant density was 2.5 SNPs per 1-kb and can well tag all currently known common variation within this region. Among the 1,039 SNPs, there were 516 SNPs with *P*<0.05 and 124 SNPs with *P*<0.0001, respectively (Figure 1A, Table S1). Remarkably, the *P* levels of 44 SNPs located in *FCRL3-CD5L* gene region were less than 1.00×10^−7^ in the initial scan cohorts (Figure 1A, Table S1, and Figure S1A).

To investigate the independent variant associated with GD on 1q21.1, we carried out the forward logistic regression analysis for the 124 SNPs with *P*<0.0001 using R statistics packages and the results displayed that rs3761959 was an independent variant in the original scan cohort. Next, the two locus logistic regression analysis was performed to confirm the independent variant. As shown in Figure 1B, rs3761959 selected as the best SNP on 1q21.1, was put individually into the regression models, and all other markers were sequentially added to see if a second locus could improve the model. The two locus logistic regression results in the original scan manifested that none of 1,031 SNPs (except 7 SNPs with high LD with rs3761959, r^2^≥0.99, Figure S1A) improved the model with rs3761959 at the *P*<0.001 level (Figure 1B). Conversely, the majority of SNPs, except for 50 SNPs with significant *P* value with the GD risk (*P*<2.30×10^−6^; Figure S1B) and within a high LD region (r^2^>0.70; Figure S1A), were improved by adding rs3761959 (Figure 1B). The logistic regression analysis could not pinpoint which SNP was the independent variant among a cluster of SNPs in a high LD block.

To further determine the most significant variant correlated with the *FCRL1-5* expression within 1q21.1 region, we carried out the cis-eQTL analysis for the 516 SNPs with *P*<0.05 in the GWAS scan cohort (Figure 1A, Table S1). From the cis-eQTL analysis using transcriptome data from about 400 lymphoblastoid cell lines [25], we found that the expression level of *FCRL3* was associated with a cluster of SNPs, with the strongest signal at rs7528684 (*P = *3.38×10^−11^ for probeset 231093_at; Figure 1C). Other three SNPs (rs2210913, rs3761959, and rs945635), which were all in high LD with rs7528684 (r^2^ = 1 in our data, Figure S1A), also exhibited the significant correlation with *FCRL3* expression (*P*<1.00×10^−10^ for probeset 231093_at; Figure 1C). Furthermore, the four SNPs also showed the eQTL effect on the expression of *FCRL5* (*P*<5.77×10^−8^ for probeset 224405_at; Figure 1C). Nonetheless, among a cluster of SNPs with the association with *FCRL5* expression, the strongest signal was located at rs6679793 (*P* = 5.90×10^−18^ for probeset 224405_at; Figure 1C), which was not in high LD with any SNP associated with the *FCRL3* expression and was weakly associated with GD in the original GWAS scan, in *FCRL5* (r^2^<0.12, *P*_GWAS_ = 0. 0252; Figure 1A, Figure S2, and Table S1).

Additionally, we inspected a cis-eQTL database assessing the transcriptome of circulating monocytes from 1,490 individuals [26] and found rs7528684 was correlated with *FCRL3* expression level (*P* = 1.41×10^−8^; Figure 1D). Also, two SNPs (rs6681271 and rs7522061), which were in high LD with rs7528684 and rs3761959 (r^2^ = 0.99, Figure S1A), displayed the high correlation with FCRL3 (*P* = 1.89×10^−8^ and 1.65×10^−8^; Figure 1D).

Ultimately, we also inspected a cell type–specific cis-eQTL database relevant to immunity and inflammation in purified B-cell and monocyte populations [27]. Of note, in the both-cis dataset, among a cluster of SNPs correlated with the expression of *FCRL3*, rs7528684 displayed higher correlation in the B cell (*P* = 7.43×10^−33^ for nuID KV7kDSLO4uggquLXB4 and *P* = 5.93×10^−26^ for nuID 67unrLpPnjv_uzOezU; Figure 1E) than that in the mono-cell (*P* = 2.35×10^−6^ for nuID KV7kDSLO4uggquLXB4 and *P = *0.0001 for nuID 67unrLpPnjv_uzOezU; Figure 1E). In the B cell-cis dataset, rs7528684 also manifested the highest correlation with *FCRL3* expression (*P = *2.37×10^−30^ for nuID ZUclXxUi6VEoBJeRT8; Figure 1F).

To confirmed the cis-eQTL analysis, We then evaluated allele-specific effects for rs3761959 and rs7528684 on the mRNA expression of *FCRL3* gene in PBMCs from 95 individuals and the result revealed both genotypes were correlated with the expression levels of *FCRL3* (*P_ANOVA_* = 0.0009) (Figure 1G). We then detected the expression of *FCRL3* in distinct PBMC populations. Although *FCRL3* was expressed in all subsets of PBMCs, there were higher expression levels of *FCRL3* in CD19^+^ B cells and CD8^+^ T subsets than those in CD4^+^ T subsets and CD14^+^ monocytes (Figure 1H). More specifically, both risk alleles A of rs3761959 and C of rs7528684 can significantly up-regulate the mRNA level of *FCRL3* in all subsets of PBMCs, especially in CD19^+^ B cells and CD8^+^ T subsets isolated from 95 healthy volunteers (*P_ANOVA_*: 0.0001 in CD4^+^ T subsets, 1.72×10^−5^ in CD8^+^ T subsets, 0.0122 in CD14^+^ monocytes, and 9.85×10^−7^ in CD19^+^ B cells, respectively; Figure 1H).

### The Association Analysis in the Replication and Combined Cohort

**Figure 2:**
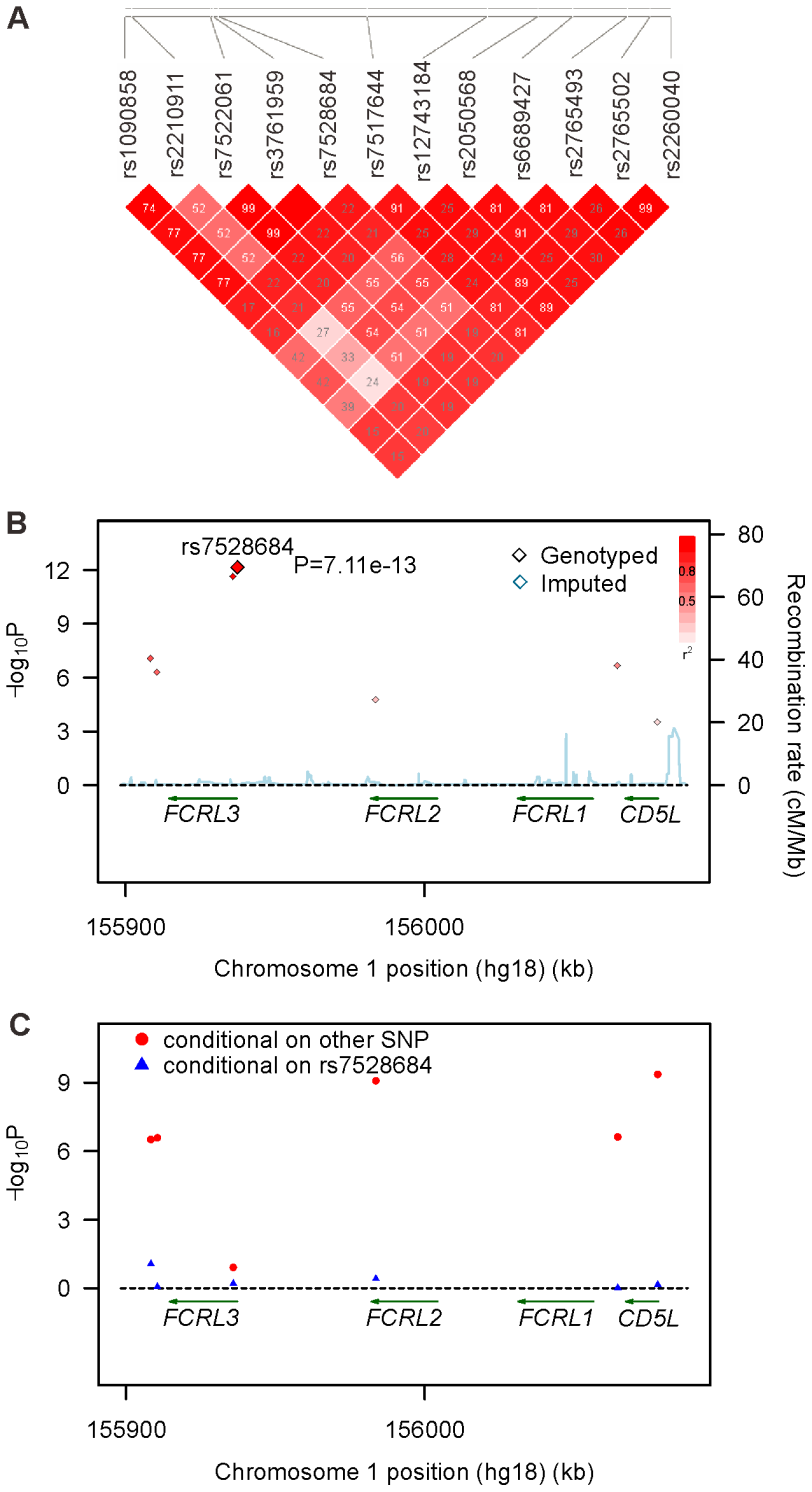
Regional plots of association results and logistic regression analysis in the combined population at 1q21.1. Panel A shows the linkage disequilibrium block analysis for the 12 SNPs with *P_GWAS_* <0.0001, which can be tagged by seven replicated SNPs in the combined population by using haploview software 4.2. Panel B shows the GD association of seven replicated SNPs in the combined population. The color of each genotyped SNP spot reflects its r^2^ with the top SNP within each association locus shown as a large red diamond, and smaller values changing from red to white. Genetic recombination rates are shown in cyan. Genetic recombination rates, estimated using the 1000 Genomes pilot 1 CHB and JPT samples, are showing cyan. Physical positions are based on NCBI build 36. Panel C shows the two locus logistic regression results for seven SNPs at 1q21.1 in the combined population. The SNPs were improved by adding rs7528684 were shown in red points; whileas, the SNPs improved the model with rs7528684 were showed in blue triangles.

Among the 124 SNPs with *P*<0.0001 in *FCRL3-CD5L* gene region, 11 SNPs were genotyped and 113 SNPs were imputed in the initial scan (Figure 1A, Table S1, and Figure S1A). Next, six genotyped SNPs tagging 11 genotyped SNPs with *P*<0.0001 and one imputed SNP (rs7528684), which were all related to *FCRL3* expression, were genotyped in the second cohort (Figure 2A). After quality control, the most significant association signal was observed at rs7528684 in 3,655 patients with GD and 3,385 controls (allele frequencies *P*_replication_ = 5.44×10^−7^, OR = 1.19, 95%CI = 1.11–1.27; and genotype distributions *P*_replication_ = 3.40×10^−6^, TC: OR = 1.17, 95%CI = 1.06–1.30, CC: OR = 1.42, 95%CI = 1.23–1.63; Table 1). Concordantly, among the seven SNPs genotyped for replication, rs7528684 displayed the highest significance in the combined datasets with 5,107 GD cases and 4,853 control individuals (allele frequencies *P*_combined_ = 7.11×10^−13^, OR = 1.23, 95%CI = 1.16–1.30; and genotype distributions *P*_combined_ = 4.87×10^−12^, TC: OR = 1.20, 95%CI = 1.09–1.31, CC: OR = 1.53, 95%CI = 1.36–1.71; Table 1; Figure 2B). Meanwhile, rs3761959 also met the genome-wide association level in the combined cohort (allele frequencies *P*_combined_ = 2.27×10^−12^, OR = 1.22, 95%CI = 1.16–1.30; and genotype distributions *P*_combined_ = 1.60×10^−12^, GA: OR = 1.19, 95%CI = 1.09–1.30, AA: OR = 1.51, 95%CI = 1.35–1.70; Table 1; Figure 2B). In addition, the forward and two-locus logistic regression analysis in the combined population demonstrated that rs7528684 could not improve the model with rs3761959 and rs3761959 also could not improve the model with rs7528684 (Figure 2C).

**Table 1:** The association results of seven SNPs on 1q23.1 in the combined populations.

SNP		GWAS (1,442 vs 1,468)				Replication (3,655 vs 3,385)				Combined (5,107 vs 4,853)			
Chr. Position	Allele/	Cases	Control			Cases	Control			Cases	Control		
Gene region	Genotype	N (%)	N (%)	OR (95% CI)	*P*	N (%)	N (%)	OR (95% CI)	*P*	N (%)	N (%)	OR (95% CI)	*P*
rs10908583	C	1,451 (50.3)	1,642 (55.9)	1.00 (ref)		3,648 (53.9)	3,717 (50.8)	1.00 (ref)		5,160 (50.7)	5,279 (54.5)	1.00 (ref)	
157641683	T	1,433 (49.7)	1,294 (44.1)	1.25 (1.13–1.39)	2.49×10^−5^	3,122 (46.1)	3,593 (49.2)	1.13 (1.06–1.21)	0.0003	5,012 (49.3)	4,403 (45.5)	1.16 (1.10–1.23)	8.55×10^−8^
*FCRL3/*	CC	385 (26.7)	463 (31.5)	1.00 (ref)		970 (28.7)	930 (25.4)	1.00 (ref)		1,313 (25.8)	1,431 (29.6)	1.00 (ref)	
FCRL4	CT	681 (47.2)	716 (48.8)	1.15 (0.97–1.36)		1,708 (50.5)	1,857 (50.8)	1.13 (1.01–1.27)		2,534 (49.8)	2,417 (49.9)	1.14 (1.04–1.25)	
	TT	376 (26.1)	289 (19.7)	1.58 (1.29–1.94)	6.76×10^−5^	707 (20.9)	868 (23.7)	1.28 (1.12–1.46)	0.0014	1,239 (24.4)	993 (20.5)	1.36 (1.22–1.52)	5.24×10^−7^
rs2210911	A	1,662 (57.6)	1,849 (63.0)	1.00 (ref)		4,112 (60.7)	4,237 (58.0)	1.00 (ref)		5,891 (57.9)	5,948 (61.4)	1.00 (ref)	
157643867	G	1,222 (42.4)	1,087 (37.0)	1.25 (1.13–1.39)	4.03×10^−5^	2,658 (39.3)	3,073 (42.0)	1.12 (1.05–1.20)	0.0008	4,281 (42.1)	3,734 (38.6)	1.16 (1.09–1.23)	4.98×10^−7^
*FCRL3/*	AA	495 (34.3)	586 (39.9)	1.00 (ref)		1,244 (36.8)	1,233 (33.7)	1.00 (ref)		1,727 (34.0)	1,827 (37.7)	1.00 (ref)	
FCRL4	AG	672 (46.6)	677 (46.1)	1.14 (0.96–1.36)		1,624 (48.0)	1,771 (48.5)	1.1 (0.99–1.22)		2,437 (47.9)	2,294 (47.4)	1.12 (1.03–1.23)	
	GG	275 (19.1)	205 (14.0)	1.56 (1.28–1.92)	0.0001	517 (15.3)	651 (17.8)	1.27 (1.1–1.46)	0.0033	922 (18.1)	720 (14.9)	1.35 (1.20–1.52)	2.31×10^−6^
rs3761959	G	1,579 (54.8)	1,817 (61.9)	1.00 (ref)		4,009 (59.2)	4,036 (55.2)	1.00 (ref)		5,607 (55.1)	5,815 (60.1)	1.00 (ref)	
157669278	A	1,305 (45.2)	1,119 (38.1)	1.34 (1.21–1.49)	5.77×10^−8^	2,761 (40.8)	3,274 (44.8)	1.18 (1.1–1.26)	1.48×10^−6^	4,565 (44.9)	3,867 (39.9)	1.22 (1.16–1.30)	2.27×10^−12^
*FCRL3*	GG	451 (31.3)	565 (38.5)	1.00 (ref)		1,178 (34.8)	1,109 (30.3)	1.00 (ref)		1,558 (30.6)	1,741 (36)	1.00 (ref)	
intron 3	GA	677 (46.9)	687 (46.8)	1.18 (1–1.38)		1,653 (48.8)	1,818 (49.7)	1.17 (1.05–1.3)		2,491 (49.0)	2,333 (48.2)	1.19 (1.09–1.30)	
	AA	314 (21.8)	216 (14.7)	1.59 (1.28–1.97)	2.10×10^−7^	554 (16.4)	728 (19.9)	1.4 (1.22–1.6)	9.07×10^−6^	1,037 (20.4)	767 (15.8)	1.51 (1.35–1.70)	1.60×10^−12^
rs7528684	T	1,571 (54.9)	1,806 (62.0)	1.00 (ref)		4,023 (59.4)	4,039 (55.3)	1.00 (ref)		5,610 (55.2)	5,829 (60.2)	1.00 (ref)	
157670816	C	1,291 (45.1)	1,106 (38.0)	1.34 (1.21–1.49)	6.59×10^−8^	2,747 (40.6)	3,271 (44.7)	1.19 (1.11–1.27)	5.44×10^−7^	4,562 (44.8)	3,853 (39.8)	1.23 (1.16–1.30)	7.11×10^−13^
*FCRL3/*	TT	449 (31.4)	563 (38.7)	1.00 (ref)		1,187 (35.1)	1,113 (30.5)	1.00 (ref)		1,562 (30.7)	1,750 (36.1)	1.00 (ref)	
*FCRL2*	TC	673 (47.0)	680 (46.7)	1.23 (1.05–1.45)		1,649 (48.7)	1,813 (49.6)	1.17 (1.06–1.30)		2,486 (48.9)	2,329 (48.1)	1.2 (1.09–1.31)	
	CC	309 (21.6)	213 (14.6)	1.82 (1.47–2.25)	2.61×10^−7^	549 (16.2)	729 (19.9)	1.42 (1.23–1.63)	3.40×10^−6^	1,038 (20.4)	762 (15.7)	1.53 (1.36–1.71)	4.87×10^−12^
rs7517644	A	2,388 (82.8)	2,568 (87.5)	1.00 (ref)		5,762 (85.1)	6,130 (83.9)	1.00 (ref)		8,502 (83.6)	8,306 (85.8)	1.00 (ref)	
157717028	G	496 (17.2)	368 (12.5)	1.45 (1.25–1.68)	5.60×10^−7^	1,008 (14.9)	1,180 (16.1)	1.1 (1–1.21)	0.0409	1,670 (16.4)	1,376 (14.2)	1.19 (1.1–1.28)	1.70×10^−5^
*FCRL2*	AA	984 (68.2)	1126 (76.7)	1.00 (ref)		2,459 (72.6)	2,568 (70.3)	1.00 (ref)		3,547 (69.7)	3,573 (73.8)	1.00 (ref)	
intron 10	AG	420 (29.1)	316 (21.5)	1.52 (1.28–1.80)		844 (24.9)	994 (27.2)	1.13 (1.01–1.26)		1,408 (27.7)	1,160 (24.0)	1.22 (1.12–1.34)	
	GG	38 (2.6)	26 (1.8)	1.67 (1.01–2.77)	1.97×10^−6^	82 (2.4)	93 (2.5)	1.09 (0.8–1.47)	0.0842	131 (2.6)	108 (2.2)	1.22 (0.94–1.58)	4.06×10^−5^
rs2765493	A	1,630 (56.5)	1,873 (63.8)	1.00 (ref)		4,032 (59.6)	4,198 (57.4)	1.00 (ref)		5,819 (57.2)	5,891 (60.8)	1.00 (ref)	
157798000	G	1,254 (43.5)	1,063 (36.2)	1.36 (1.22–1.51)	1.76×10^−8^	2,738 (40.4)	3,112 (42.6)	1.09 (1.02–1.17)	0.0108	4,353 (42.8)	3,791 (39.2)	1.16 (1.1–1.23)	2.16×10^−7^
*CD5L/*	AA	473 (32.8)	590 (40.2)	1.00 (ref)		1,209 (35.7)	1,210 (33.1)	1.00 (ref)		1,682 (33.1)	1,795 (37.1)	1.00 (ref)	
FCRL1	AG	684 (47.4)	693 (47.2)	1.23 (1.05–1.45)		1,614 (47.7)	1,778 (48.6)	1.1 (0.99–1.22)		2,455 (48.3)	2,301 (47.5)	1.14 (1.04–1.24)	
	GG	285 (19.8)	185 (12.6)	1.92 (1.54–2.4)	4.17×10^−8^	562 (16.6)	667 (18.2)	1.19 (1.03–1.36)	0.0377	949 (18.7)	745 (15.4)	1.36 (1.21–1.53)	1.27×10^−6^
rs2260040	A	2,382 (82.6)	2,552 (86.9)	1.00 (ref)		5,706 (84.3)	6,094 (83.4)	1.00 (ref)		8,460 (83.2)	8,235 (85.1)	1.00 (ref)	
157811392	G	502 (17.4)	384 (13.1)	1.4 (1.21–1.62)	4.41×10^−6^	1,064 (15.7)	1,216 (16.6)	1.07 (0.98–1.17)	0.1403	1,712 (16.8)	1,447 (14.9)	1.15 (1.07–1.24)	0.0003
*CD5L*	AA	980 (68.0)	1,112 (75.7)	1.00 (ref)		2,411 (71.2)	2,538 (69.4)	1.00 (ref)		3,513 (69.1)	3,512 (72.5)	1.00 (ref)	
intron 1	AG	422 (29.3)	328 (22.3)	1.46 (1.23–1.73)		884 (26.1)	1,018 (27.9)	1.09 (0.98–1.22)		1,434 (28.2)	1,211 (25)	1.18 (1.08–1.29)	
	GG	40 (2.8)	28 (1.9)	1.62 (0.99–2.65)	1.67×10^−5^	90 (2.7)	99 (2.7)	1.04 (0.78–1.4)	0.2494	139 (2.7)	118 (2.4)	1.18 (0.92–1.51)	0.0007

SNP: single nucleotide polymorphism, N-number, OR- odds ratio for the minor allele, 95% CI- 95% confidence interval. We report a 1-df test P-value for allelic effects and a 2-df test P-value for genotype effects.

Because multiple SNPs may act in combination to increase the risk of disease, haplotypes of the SNPs in the combined population were investigated and their frequencies in the GD and control groups were compared. The results displayed that seven haplotypes with a frequency of more than 4% were formed from seven SNPs and accounted for about 95% of all haplotypes (Table 2). Four of seven haplotypes exhibited significantly higher frequencies among individuals with GD than the control group. As shown in Table 2, the haplotype TGACGGG manifested the highest statistical difference (*P* = 1.15×10^−5^, OR = 1.20, 95%CI = 1.11–1.30; Table 2), followed by haplotypes TGACAGA and TGACAAA (*P* = 0.0214, OR = 1.10, 95%CI = 1.01–1.20; and *P* = 0.0044, OR = 1.18, 95%CI = 1.05–1.33, respectively; Table 2). In contrast, haplotypes CAGTAAA and TGGTAAA were more frequently observed in controls than in patients with GD (*P* = 4.73×10^−8^, OR = 0.86, 95%CI = 0.81–0.91; and *P* = 0. 0262, OR = 0.87, 95%CI = 0.76–0.98, respectively; Table 2). Notably, all the risk haplotypes of GD contained the risk allele A of rs3761959 and risk allele C of rs7528684 and all the protected haplotypes of GD contained the protected allele G of rs3761959 and protected allele T of rs7528684 (Table 2).

**Table 2:** Frequencies of the haplotypes on 1q23.1 in the combined population.

rs10908583	rs2210911	rs3761959	rs7528684	rs7517644	rs2765493	rs2260040	control N(%)	case N(%)	*P*	OR (95% CI)
**T**	**G**	**A**	**C**	**G**	**G**	**G**	**1199 (12.4)**	**1476 (14.5)**	**1.15**×**10^−5^**	**1.20 (1.11–1.30)**
**T**	**G**	**A**	**C**	**A**	**G**	**A**	**1230 (12.7)**	**1405 (13.8)**	**0.0214**	**1.10 (1.01–1.20)**
**T**	**G**	**A**	**C**	**A**	**A**	**A**	**543 (5.6)**	**669 (6.6)**	**0.0044**	**1.18 (1.05–1.33)**
T	A	A	C	A	G	A	562 (5.8)	633 (6.2)	0.2154	1.08 (0.96–1.21)
C	A	G	T	A	G	A	454 (4.7)	468 (4.6)	0.7677	0.98 (0.86–1.12)
**T**	**G**	**G**	**T**	**A**	**A**	**A**	**534 (5.5)**	**490 (4.8)**	**0.0262**	**0.87 (0.76–0.98)**
**C**	**A**	**G**	**T**	**A**	**A**	**A**	**4668 (48.2)**	**4511 (44.3)**	**4.73**×**10^−8^**	**0.86 (0.81–0.91)**

Bold letters indicate those haplotypes with significant differences between GD and normal subjects. All data shown here are haplotypes whose frequencies are more than 2%.

**Figure 3:**
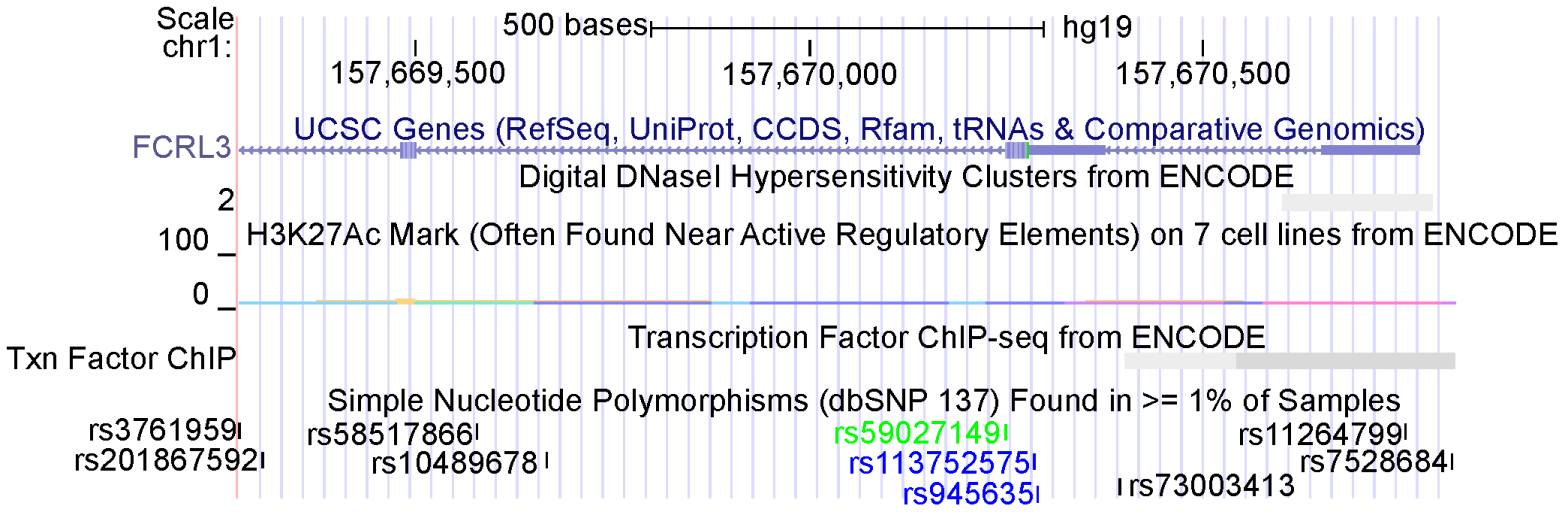
The epigenetic analysis from the ENCODE database. The chromosome region containing rs7528684 displayed no DNaseI hypersensitivity and can bind the transcription factor. However, the region harboring rs3761959 shows no binding with the transcription factor.

Ultimately, we used the ENCODE databases of epigenetic study to narrow down the candidate regulatory regions and polymorphisms (http://genome.ucsc.edu/ENCODE/) [29]. The data from ENCODE manifested the chromosome region containing rs7528684 was without a DNaseI hypersensitivity, however, can bind the transcription factor (Figure 3). Also, we found the risk allele C of rs7528684 can bind the transcription factor NF-KB utilizing the Searching Transcription Factor Binding Sites (TFSEARCH, ver 1.3) (score: 96.9) [30]. The previous study also found rs7528684 could affect the *FCRL3* expression in the luciferase assay [16]. Whereas, the chromosome region containing rs3761959 could not bind the transcription factors (Figure 3), suggesting the association between rs3761959 and GD because of its high LD with rs7528684.

The false positive report probability (FPRP) of the SNPs with significant association to GD in the combined Chinese Han cohort was also analyzed. In the present study, the FPRP value was calculated for each genetic variant using the assigned prior probability range, the statistical power to detect an odds ratio of 1.5, and detected odds ratios and *P* values. As shown in Table 3, among the seven genetic variants, the FPRP values of five SNPs were below 0.2 for the prior probability from 0.25 to 0.00001, which was a relatively high prior probability range. In addition, the FPRP values for rs7528684 were still the lowest even for a prior probability of 0.00001 (Table 3). Interestingly, the case-control association study for these seven SNPs with significant differences in allele frequencies between the 5,107 patients with GD and 4,853 control individuals has 100% statistical power to detect a SNP with an α level equal to their reported *P* value, corresponding to relative risks of 1.5 for GD (Table 3).

**Table 3:** False positive report probability (FPRP) values for seven SNPs with significant difference between 5,300 patients with GD and 4,916 health individuals.

SNP	Odds ratio (95% CI)	Reported *p*-Value	Statistical power under recessive model^a^	Prior probability					
				0.25	0.1	0.01	0.001	0.0001	0.00001
rs10908583	1.16 (1.10–1.23)	8.55×10^−8^	1.0000	**2.59**×**10^−7^**	**7.77**×**10^−7^**	**8.54**×**10^−6^**	**8.62**×**10^−5^**	**0.0009**	**0.0086**
rs2210911	1.16 (1.09–1.23)	4.98×10^−7^	1.0000	**1.32**×**10^−6^**	**3.95**×**10^−6^**	**4.35**×**10^−5^**	**0.0004**	**0.0044**	**0.0420**
rs3761959	1.22 (1.16–1.30)	2.27×10^−12^	1.0000	**6.07**×**10^−12^**	**1.82**×**10^−11^**	**2.00**×**10^−10^**	**2.02**×**10^−9^**	**2.02**×**10^−8^**	**2.02**×**10^−7^**
rs7528684	1.23 (1.16–1.30)	7.11×10^−13^	1.0000	**1.83**×**10^−12^**	**5.48**×**10^−12^**	**6.03**×**10^−11^**	**6.08**×**10^−10^**	**6.09**×**10^−9^**	**6.09**×**10^−8^**
rs7517644	1.19 (1.10–1.28)	1.70×10^−5^	1.0000	**4.96**×**10^−5^**	**0.0001**	**0.0016**	**0.0162**	**0.1418**	0.6230
rs2765493	1.16 (1.10–1.23)	2.16×10^−7^	1.0000	**5.69**×**10^−7^**	**1.71**×**10^−6^**	**1.88**×**10^−5^**	**0.0002**	**0.0019**	**0.0186**
rs2260040	1.15 (1.07–1.24)	0.0003	1.0000	**0.0009**	**0.0026**	**0.0275**	0.2218	0.7405	0.9661

a Statistical power is the power to detect an odds ratio of 1.5 for the homozygotes with the rare genetic variant, with an α level equal to the reported p-Value. FPRP values below 0.2 are in bold face.

## Discussion

Our refined association study of the SNPs on 1q21.1 region verified that *FCRL3* was the susceptibility gene for GD in the Chinese Han population. Moreover, the logistic regression analysis revealed that 8 SNPs including rs3761959 may be the most likely susceptibility variant. Cis-eQTL analysis from three databases indicated that the most significant signals correlated to the expression of *FCRL3* were located at a cluster of SNPs including rs3761959 and rs7528684. In the combined population analysis, the risk haplotypes containing the risk allele A of rs3761959 and risk allele C of rs7528684 were associated with the predisposition of GD. Furthermore, the risk allele A of rs3761959 and risk allele C of rs7528684 increased *FCRL3* expression whether in PBMCs or in its subsets, especially in CD19^+^ B cells and CD8^+^ T subsets. However, only rs7528684 can bind the NF-KB transcription factor to affect the *FCRL3* expression. Intriguingly, the FPRP value for SNP rs7528684 was very low for the prior probability range and was quite robust even for low prior probabilities. These results suggested that rs7528684 in the promoter of *FCRL3* was associated with GD etiology in the combined Chinese Han population.

The first reported variant about the association of *FCRL3* with GD was rs7528684 located at position –169 in promoter of *FCRL3* gene in a Japanese population (*P* = 7.4×10^−5^) [16], which was confirmed by the later study in 1,056 UK patients with GD and 864 controls (*P* = 0.024) [31]. Our two-stage GWAS analysis also confirmed rs7528684 was associated with GD in Chinese Han population. Although there were two negative conclusions regarding association of rs7528684 in *FCRL3* with GD in two small samples studies: one was in a Chinese population with 436 cases and 316 controls [32], and the other was in a UK population based on 625 cases and 490 controls (Table S2) [33], we still regarded SNP rs7528684 as a susceptibility GD locus in *FCRL3* region in the Chinese Han population. Meanwhile, all of the risk haplotypes of GD contained the risk allele C of rs7528684, which can increase *FCRL3* expression both from cis-eQTL analysis and from the real-time PCR results.

Later, WTCCC in an analysis including 2,500 UK GD cases and 2,500 controls found an association at rs3761959 (a perfect proxy of rs7528684, r^2^ = 1 in our data; Figure 2A) with GD (*P* = 0.0094; Table S2) [19]. Also, rs3761959 showed the significant association with GD in our two-stage GWAS analysis and could affect the expression of *FCRL3* from our cis-eQTL analysis and real-time PCR. Noteworthy, in the WTCCC study, a stronger association was found with rs11264798 (in high LD with rs7528684 in our data, r^2^ = 0.99, *P* = 1.6×10^−5^; Table S2), located in the intron 8 of *FCRL3* [19]. More recently, the WTCCC genotyped 743 SNPs across *FCRL3* in 7,894 control samples and about 2,000 GD subjects to define the causal GD-associated SNPs using Bayes theorem [34]. Unfortunately, the fine mapping data from WTCCC failed to refine the signal in *FCRL3* due to the 95% credible set containing 114 SNPs, albeit the top SNP, rs11264798, accounting for 7% of the posterior weight (Table S2) [34]. The imputation analysis manifested that SNP rs11264798 was also associated with GD in our initial scan cohort (*P_GWAS_* = 4.29×10^−7^; Table S2). At the same time, we compared our data with those in the study by Simmonds et al that also fine-mapped the *FCRL5* region in a UK population and found none of 11 SNPs (excluding SNP rs3900700 because of MAF <0.01 in Chinese Han population) showed the association with GD in the Chinese Han population at the *P*<0.05 level (Table S2). Above results suggests that rs7528684 should be the likely *FCRL* etiological variant in our Chinese Han GD population, albeit the possible roles for the proxy of rs3761959 or rs11264798 maybe exist.

Although the three previous eQTL studies were all from the Caucasians rather than from the Han Chinese population, however, the LD analysis between the two populations displayed a similar LD block harboring *CD5L* and all three significant SNPs located in *FCRL3* (rs11264798, rs3761959 and rs7528684) falling in the same LD block (Figure S3). Interestingly, there were two LD blocks and the five genes (*FCRL1-5)* were located in the first LD block in the HapMap CEU populations (Figure S3A). Whilst, among three LD blocks in CHB and JPT populations, two genes (*FCRL5-4*) and three genes (*FCRL3-1)* were located in two different LD blocks, respectively (Figure S3B).

*FCRL3* gene is one of five *FCRL* genes that shares significant structural homology to classical receptors for immunoglobulin constant chains (Fc receptors) [35] and encodes a protein containing an immunoreceptor-tyrosine activation motif and immunoreceptor-tyrosine inhibitory motif in its cytoplasmic domain [36]. *FCRL3* is highly expressed in lymphoid organs, particular strongly on the surface of the B-cells, but also on that of the T-cells [16]. Among B-cell subsets, *FCRL3* molecule is present on mature, germinal center, memory, plasma cells, and bone marrow immature B cells suggesting for its key role in the development, maturation, and function of B-lymphocytes [37]. The pathogenic activation of *FCRL3* expression is suggested to lead to the down-regulation of B cell receptor-mediated signaling, incomplete induction of anergy and deletion in autoreactive B-cells, and, finally, to breakdown of B-cell tolerance [38]. Presence of *FCRL3* was also demonstrated on the surface of a subset of Treg cells characterized by lower relative response to antigenic stimulation and reduced suppressor activity [39]. In the original report, rs7528684 was suggested to have functional significance as the disease-associated C allele increased affinity for NF-KB transcription factor and showed enhanced transcription rate in luciferase assay [16]. In our study, CD19^+^ B-cells had the highest expression of *FCRL3* among the four subsets of PBMCs and the risk allele C of rs7528684 was significantly correlated with elevated mRNA expression level of *FCRL3* mainly in CD19^+^ B cells and CD8^+^ T subsets, secondly in CD4^+^ T subsets and CD14^+^ monocytes. Moreover, our epigenetic analysis from ENCODE database and TFSEARCH software analysis also manifested that the risk allele C of rs7528684 could bind to NF-KB transcription factor. Therefore, we proposed that *FCRL3* gene and its proxy SNP rs7528684 can be involved in the pathogenesis of GD by excessive inhibiting B cell receptor signaling and the impairment of suppressing function of Tregs.

In summary, our study provided the unequivocal evidence that *FCRL3* was the susceptibility gene of GD and its proxy SNP rs7528684 may be the etiology variant to predispose to GD in Chinese Han population.

## Supporting Information

Figure S1 **Regional plots of association results and linkage disequilibrium structure of 58 SNPs.** Panel A shows the linkage disequilibrium (LD) structure for the 8 SNPs with high LD with rs3761959 in the first LD block and 50 SNPs that could not be improved in the model with rs3761959 in the second LD block in the GWAS samples. Panel B shows the GD association of 58 SNPs with *P*<2.30×10^−6^the linkage disequilibrium (LD) structure for the 51 SNPs in the GWAS samples. The color of each genotyped SNP spot reflects its r^2^ with the top SNP within each association locus shown as a large red diamond, and smaller values changing from red to white. Genetic recombination rates are shown in cyan. Genetic recombination rates, estimated using the 1000 Genomes pilot 1 CHB and JPT samples, are showing cyan. Physical positions are based on NCBI build 36.
(TIF)

Figure S2 **The linkage disequilibrium structure of 210 SNPs including rs6679793 in the GWAS scan cohort.** The 210 SNPs contains 209 SNPs correlated to the *FCRL3* expression and rs6679793 which is the top SNP correlated to the *FCRL5* expression.
(TIF)

Figure S3 **The linkage disequilibrium structure for the region 155,744-156,152 Kb at 1q21.1 in the CEU (A) and CHB and JPT (B) population from the HapMap phase II 24 release. Coloring in the figure is according to r^2^.**
(TIF)

Table S1 **Association results of the imputed and typed SNPs in 1q21.1 region with GD in initial genome-wide scan.**
(XLS)

Table S2 **The comparison among the results from three studies on FCRL genes in 1q21.1.**
(XLS)
