# Supplementary figures and images for: A Refined Study of FCRL Genes from a Genome-Wide Association Study for Graves’ Disease
Source: PLoS One. 2013 Mar 7;8(3):e57758. doi: 10.1371/journal.pone.0057758 (PMC3591391; doi:10.1371/journal.pone.0057758)

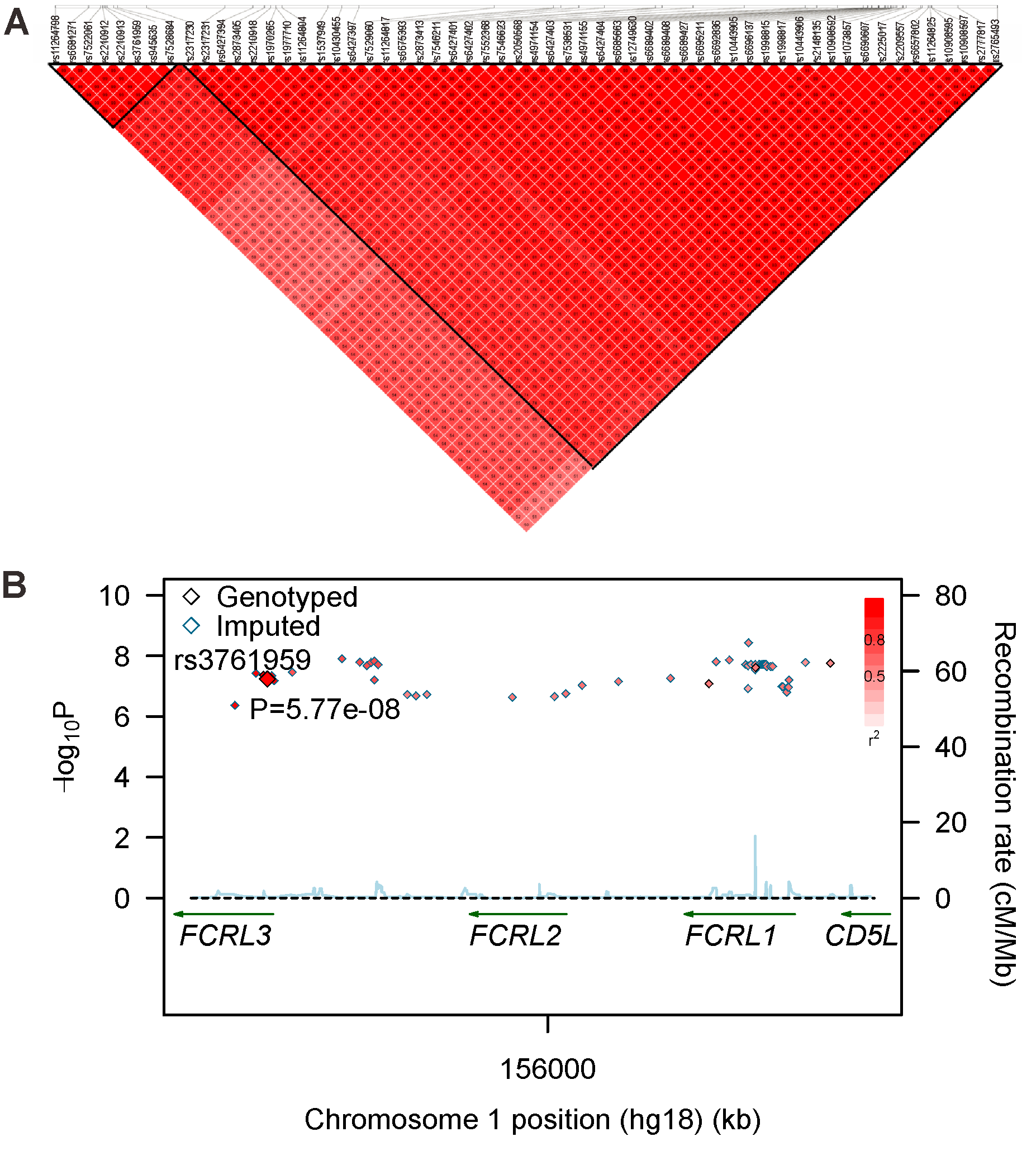

Supplement: Figure S1 — Regional plots of association results and linkage disequilibrium structure of 58 SNPs. Panel A shows the linkage disequilibrium (LD) structure for the 8 SNPs with high LD with rs3761959 in the first LD block and 50 SNPs that could not be improved in the model with rs3761959 in the second LD block in the GWAS samples. Panel B shows the GD association of 58 SNPs with P<2.30×10^−6^the linkage disequilibrium (LD) structure for the 51 SNPs in the GWAS samples. The color of each genotyped SNP spot reflects its r2 with the top SNP within each association locus shown as a large red diamond, and smaller values changing from red to white. Genetic recombination rates are shown in cyan. Genetic recombination rates, estimated using the 1000 Genomes pilot 1 CHB and JPT samples, are showing cyan. Physical positions are based on NCBI build 36. (TIF) [file pone.0057758.s001.tif]

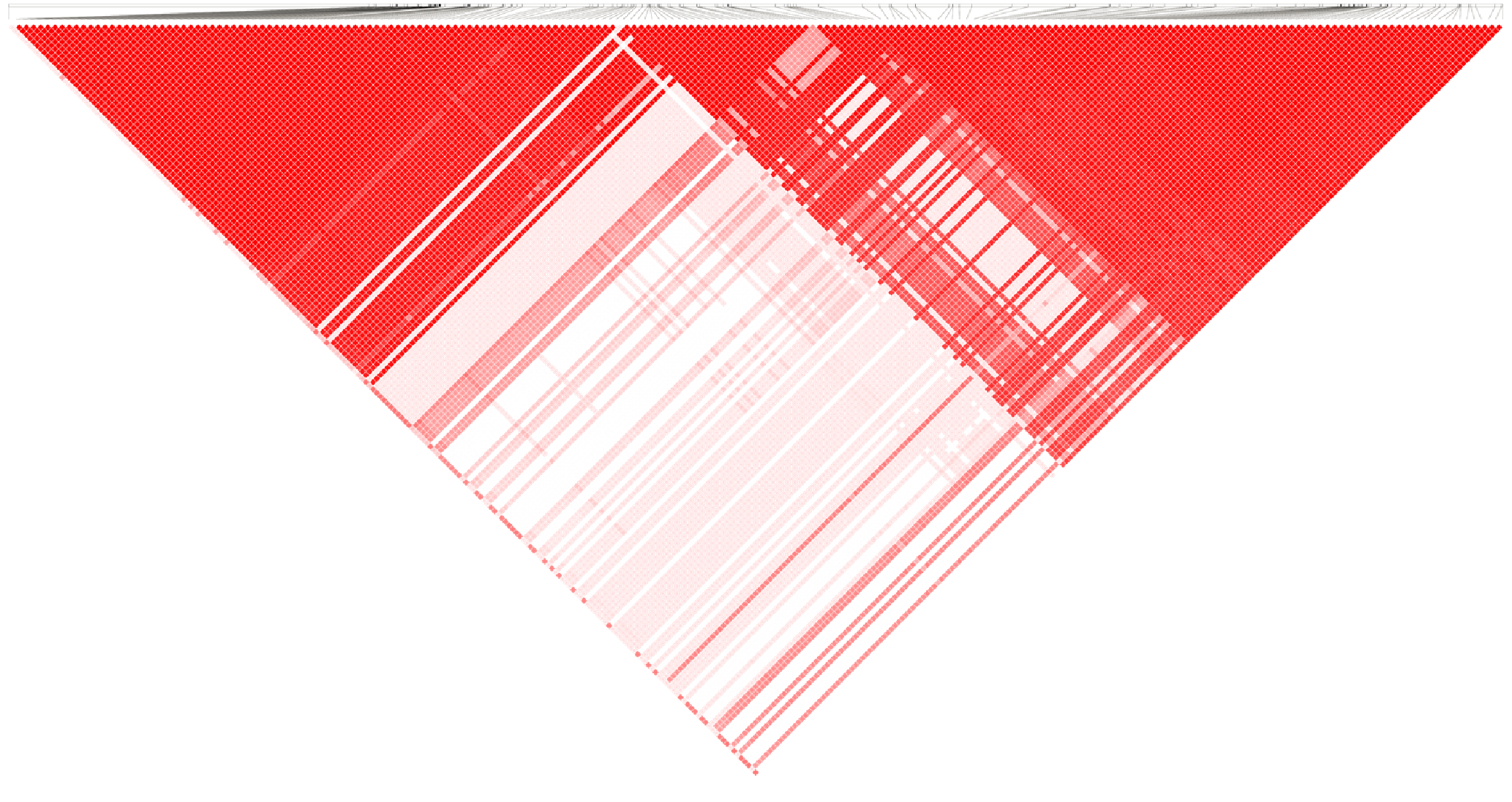

Supplement: Figure S2 — The linkage disequilibrium structure of 210 SNPs including rs6679793 in the GWAS scan cohort. The 210 SNPs contains 209 SNPs correlated to the FCRL3 expression and rs6679793 which is the top SNP correlated to the FCRL5 expression. (TIF) [file pone.0057758.s002.tif]

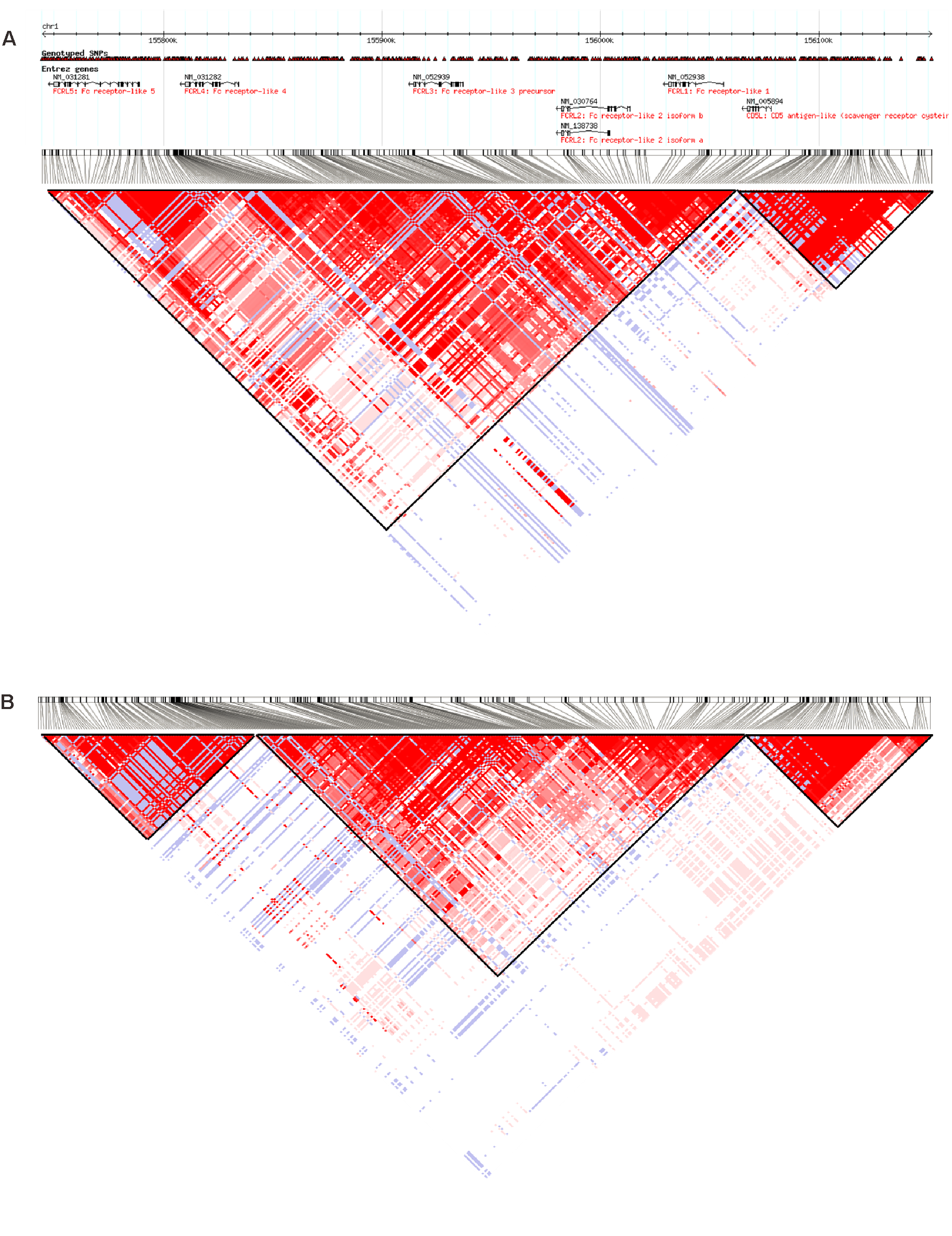

Supplement: Figure S3 — The linkage disequilibrium structure for the region 155,744-156,152 Kb at 1q21.1 in the CEU (A) and CHB and JPT (B) population from the HapMap phase II 24 release. Coloring in the figure is according to r2. (TIF) [file pone.0057758.s003.tif]
